# Attitude toward and awareness of medical-dental collaboration among medical and dental students in a university in Indonesia

**DOI:** 10.1186/s12903-019-0848-8

**Published:** 2019-07-15

**Authors:** Diah Ayu Maharani, Stacia Ariella, Intan Detrianis Syafaaturrachma, Indriasti Indah Wardhany, Armasastra Bahar, Shinan Zhang, Sherry Shiqian Gao, Chun Hung Chu, Anton Rahardjo

**Affiliations:** 1 Department of Preventive and Public Health Dentistry, Faculty of Dentistry, Universitas Indonesia, Jalan Salemba No. 4, Jakarta, 10430 Indonesia; 2 Faculty of Dentistry, Universitas Indonesia, Jakarta, Indonesia; 3 Department of Oral Medicine, Faculty of Dentistry, Universitas Indonesia, Jakarta, Indonesia; 4 Faculty of Stomatology, Kunming Medical University, Kunming, China; 5 Faculty of Dentistry, The University of Hong Kong, Hong Kong, China

**Keywords:** Medical-dental collaboration, Attitude, Awareness, Medical students, Dental students

## Abstract

**Background:**

Medical-dental collaboration expands patients’ access to health services, improves healthcare outcomes, and reduces the burden and cost of care, especially for those with chronic diseases. The aim of the present study is to investigate the attitude toward and awareness of medical-dental collaboration among medical and dental students attending the Universitas Indonesia.

**Methods:**

All medical and dental students at the Universitas Indonesia were invited to participate in a web-based questionnaire survey that contained eight questions on attitudes toward medical-dental collaboration and two questions regarding awareness of dental-medical collaborative practices. The demographic backgrounds of all participants were obtained. The chi-square test and logistic regression analysis were employed for data analysis.

**Results:**

A total of 1,432 questionnaires were distributed, and 1,137 (79%) were appropriately completed. In general, 992 (87%) students had a positive attitude toward medical-dental collaboration. Dental students had a more positive attitude than medical students (odds ratio [OR] = 2.694; *p* = 0.001), and senior students had a more positive attitude than junior students (OR = 2.271; *p* = 0.001). Most students (86%) were aware of medical-dental collaboration at the Universitas Indonesia and reported that emergency medicine, surgery, and otolaryngology were the three most common medical disciplines that entailed medical-dental collaboration. *Conclusions*: In general, the medical and dental students demonstrated positive attitudes and awareness of medical-dental collaboration at the Universitas Indonesia. Positive attitude and awareness can establish an essential foundation for fostering collaboration between medicine and dentistry, which is vital to improve resource efficiency and healthcare standards.

## Background

Oral health has both physical and psychological effects, as poor oral health is associated with considerable pain and various problems related to mastication, speaking ability, appearance, growth, social wellbeing, and quality of life [1]. Therefore, maintenance of good oral health is essential to good general health. In fact, assessment of the oral cavity and oral secretions can reveal manifestations of certain systemic diseases. Hence, early detection of oral symptoms is sometimes crucial for the diagnosis of diseases in other parts of the body [2]. Additionally, when there are indications of chronic and multimorbid pathologies, healthcare becomes more complex. Hence, interprofessional collaboration among various disciplines is vital for the clinical success of a comprehensive healthcare plan [3]. Indeed, apart from the obvious clinical benefits, proficient collaboration between medical and dental professionals also improves resource efficiency, as well as the standards, continuity, and comprehensiveness of healthcare plans by reducing duplication and gaps in services [4]. Therefore, medical-dental collaboration expands the patient’s access to health services, improves healthcare outcomes, and reduces the burden and cost of care, especially for those with chronic diseases [5].

In Indonesia, medical doctors and dentists are trained separately as distinct professionals with different responsibilities [6]. At the Universitas Indonesia, undergraduate students of the Faculty of Medicine and the Faculty of Dentistry must complete 144 credits within eight semesters to meet the criteria of the undergraduate study program. Upon completion of these credits, the medical curriculum of the clinical practice program requires the completion of 69 credits within four semesters, which is then followed by a 1-year internship program conducted by the government. During medical training, the university does not require credits from courses in dentistry. On the other hand, the dental education program (professional stage) requires the completion of 34 credits within four semesters. However, the dental curriculum requires relatively minimal basic medical training. Consequently, dentists usually focus on the diagnosis and treatment of symptoms limited to the oral cavity and may overlook other problems affecting general health. Likewise, medical doctors may fail to assess oral health problems, thereby disregarding indicators of systemic diseases [4].

Interprofessional education could increase access to health care and provide students with a foundation for collaborative practice in the community, increase awareness of their respective fields, deliver high-quality health care and promote health equity [7–10]. It follows that it is important to understand the attitude and awareness of dental and medical students with regard to interprofessional care. However, although medical-dental collaboration is essential to clinical practice, limited studies have investigated its impact in the clinical context. Moreover, no study has investigated the perspectives of medical and dental students regarding medical-dental collaboration in Indonesia. Therefore, the aim of the present study is to investigate the attitude towards and awareness about medical-dental collaboration among medical and dental students attending the Universitas Indonesia.

## Methods

### Study design and recruitment of participants

In this cross-sectional study, all medical and dental students attending the Universitas Indonesia (*N* = 1,432) were invited to participate in a questionnaire survey in November 2017. The study protocol was approved by the Research Ethics Committee, Faculty of Dentistry, Universitas Indonesia (approval no. 104/Ethical Approval/FKGUI/ix/2017), and written informed consent was obtained from all participants. A link to the web-based questionnaire was sent to the coordinators of each class, who were responsible for distributing the link and monitoring the students during completion of the survey. The survey period was limited to a period of 1 month. The questionnaire was sent out twice: the second wave of questionnaires was sent a week after the first wave, but it was only sent to students who had not yet filled in the questionnaire yet.

### Questionnaire survey

The questionnaire survey used herein was validated in a previous study [4], which had adapted it from the questions used by other published studies [11, 12]. The original questionnaire was translated into Bahasa Indonesian by the forward-backward process. For this, the questionnaire was first translated from English to Bahasa Indonesian by an independent bilingual speaker. The validity of the first Bahasa Indonesian draft of the questionnaire was discussed among the members of an expert panel. Then, the draft was translated back into English by a second independent bilingual translator. The back-translated English version was compared with the original English version by the panel members to evaluate the semantic equivalence. Based on the results of semantic equivalence, further revisions were made. A pilot test of the revised version of the questionnaire was conducted using a cohort of 20 university students to ensure the clarity and comprehensiveness of the wording.

The final questionnaire consisted of 3 components and a total of 16 questions. The purpose of the first component was to collect the demographic information of the participants, which included (1) curriculum (medicine or dentistry), (2) year of study (year 1–3 was considered as the junior year/year 4–5 was considered as the senior year), (3) age (< 21/≥ 21 years), (4) gender (male/female), (5) having a family physician (yes/no), and (6) time since last dental check-up (≤12 months/> 12 months/no regular dental check-up). The second component focused on the participants’ attitudes toward medical-dental collaboration, and it included eight “yes or no” questions. The third component investigated the students’ awareness of medical-dental collaboration in Indonesia. If the student was aware of medical-dental collaboration, a follow-up question was asked to assess the perceived links between the field of dentistry and 11 medical disciplines (i.e. emergency medicine, cardiothoracic surgery, clinical oncology, otolaryngology, family medicine, general surgery, obstetrics & gynaecology, orthopaedics & traumatology, paediatric medicine, psychiatry, and radiology) in Indonesia.

### Data analysis

The collected data were entered into an Excel file by SA and IDS and, then, cleaned and checked by another researcher (DAM) before analysis. A web-based questionnaire was adopted because it has been reported to be more cost-effective, to have a lower number of missing values and, also, to provide a higher data completeness rate than data collection with paper questionnaires [13]. IBM SPSS Statistics for Windows, version 24.0 (IBM Corporation Armonk, NY, USA) was used to conduct data analysis. Attitudes toward medical-dental collaboration were scored based on a calculation of the responses to eight attitude questions: “Yes” was assigned 1 point and “No” was assigned 0 points. The scores of all the questions were summed up as the total attitude score. The total attitude scores were categorized into three groups: negative (score, 0–2), neutral (score, 3–5), and positive (score, 6–8). Descriptive analysis of the response to each question was conducted. The chi-square test and logistic regression analysis were used to study the relationship between the dependent variables (students’ attitudes towards and awareness of medical-dental collaboration) and the independent variables (curriculum, year of study, age, gender, having a family physician, and last dental visit). The statistical significance level was set at 0.05 for all tests.

## Results

From a total of 1,432 medical and dental students from the Universitas Indonesia who were invited to participate in this study, 1,137 valid questionnaires (response rate, 79%) were collected from 579 medical students and 558 dental students. Table 1 presents the demographic information of the participating students and their attitudes towards and awareness of medical-dental collaboration. Most students (97%) agreed that “oral health is an integral part of general health,” but many did not agree that dental students should have a rotation in medicine (33%) or vice versa (34%). The majority of students (*n* = 992, 87%) had a positive attitude (score, 6–8) toward medical-dental collaboration, whereas some (*n* = 142, 13%) had an neutral attitude (score, 3–5) and three had a negative attitude (score, 0–2). Students with an neutral or negative attitude were combined into one group as “students with a fair attitude,” for the following chi-square tests and logistic regression analysis.

**Table 1:** Participants’ demographic information and attitudes and awareness toward medical-dental collaboration

Item (*N* = 1137 respondents)	n (%)
Demographic information	
Curriculum	
Medicine	579 (51)
Dentistry	558 (49)
Year of study	
Year 1–3	721 (63)
Year 4–5	416 (37)
Age, years	
≤ 20	754 (66)
> 21	383 (34)
Gender	
Male	332 (29)
Female	805 (71)
Having a family physician	
Yes	230 (20)
No	907 (80)
Last dental check-up	
< 1 year	406 (36)
≥ 1 year	21 (2)
No regular dental check-up	710 (62)
Attitude and awareness	
Dentist is a profession similar to medical practitioners	
Yes	1,113 (98)
No	24 (2)
Oral health is an integral part of general health	
Yes	1,100 (97)
No	37 (3)
Dentists should be included in electronic health record system	
Yes	1,101 (97)
No	36 (3)
Medical-dental collaboration enhances quality of patient care	
Yes	1,085 (95)
No	52 (5)
Dentist is responsible to advise patients on systemic health	
Yes	980 (86)
No	157 (14)
Physician is responsible to advise patients on oral health	
Yes	1,042 (92)
No	95 (8)
Dental students should have a rotation in medicine	
Yes	766 (67)
No	371 (33)
Medical students should have a rotation in dentistry	
Yes	752 (66)
No	385 (34)
Aware of any collaboration between dentistry and medicine	
Yes	979 (86)
No	158 (14)

Table 2 shows the results of the chi-square test, which indicate the relationships between the students’ attitudes toward medical-dental collaboration and the independent variables. The results of logistic regression confirmed that the curriculum and year of study were significant factors related to the students’ attitudes (Table 3). Overall, dental students had a more positive attitude toward medical-dental collaboration than medical students (odds ratio [OR] = 2.694; 95% confidence interval [CI] = 1.838–3.948; *p* = 0.001), and senior students (year 4–5) were more positive about the collaboration than junior students (OR = 2.271; 95% CI = 1.497–3.446; *p* = 0.005).

**Table 2:** Variables related to the students’ attitudes toward medical-dental collaboration (chi-square test)

Variable (n)	Positive attitude, n (%)	*p*
Curriculum		0.001
Medicine (579)	476 (82)	
Dentistry (558)	516 (93)	
Year of study		0.001
Year 1–3 (721)	608 (84)	
Year 4–5 (416)	384 (92)	
Age, years		0.050
≤ 20 (754)	640 (85)	
> 21 (383)	352 (92)	
Gender		0.193
Male (332)	283 (85)	
Female (805)	709 (88)	
Having a family physician		0.461
Yes (907)	788 (87)	
No (230)	204 (89)	
Last dental check-up		0.159
< 1 year (21)	20 (95)	
≥ 1 year (406)	362 (89)	
No regular dental check-up (710)	610 (86)

**Table 3:** Variables related to the students’ attitude toward medical-dental collaboration (logistic regression)

Variable	Odds ratio	95% CI	*p*
Curriculum			0.001
Medicine ^Ref^			
Dentistry	2.694	1.8381–3.948	
Year of study			0.001
Year 1–3 ^Ref^			
Year 4–5	2.271	1.497–3.446	
Constant	3.595		0.001

*CI* confidence interval, *Ref* reference

**Fig. 1:**
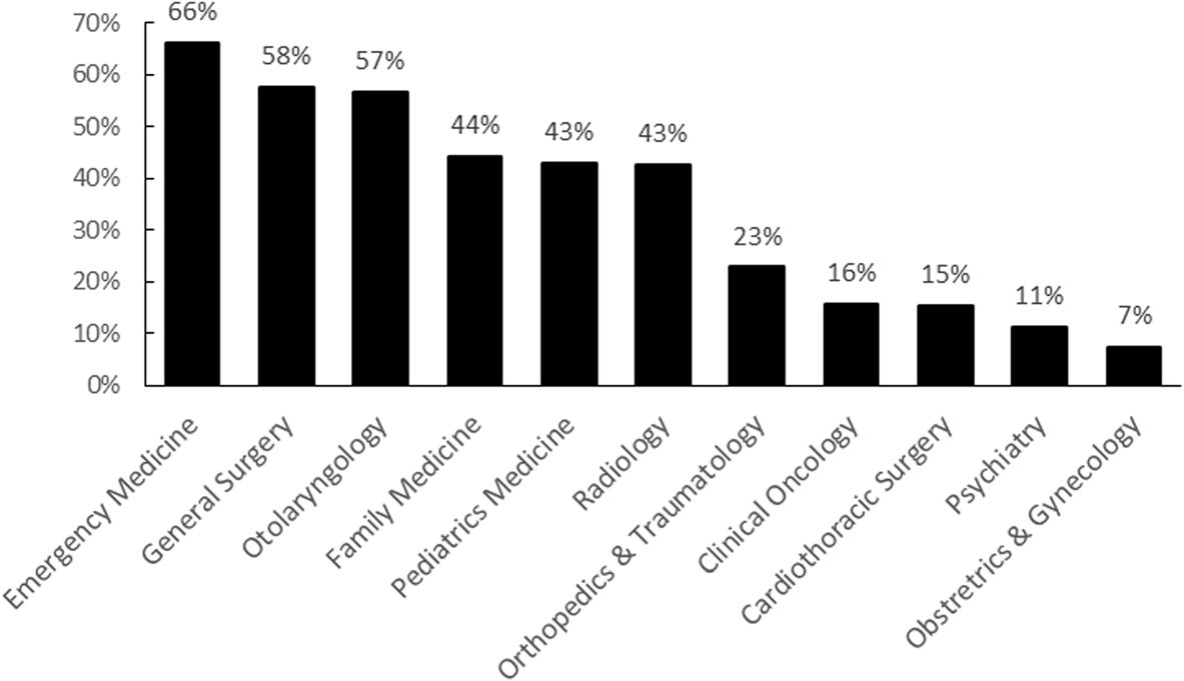
Students’ perception (% respondents) on the medical disciplines related to dentistry

Most students (86%) were aware of medical-dental collaboration (Table 1). Table 4 describes the relationships between the students’ awareness of medical-dental collaboration and the independent variables. Curriculum, year of study, age, gender, and last dental check-up experience were correlated to students’ awareness. The results of logistic regression analysis showed that dental students were more aware of medical-dental collaboration than medical students were (OR = 3.352; 95% CI = 2.243–5.010; *p* = 0.001), and senior students (year 4–5) were more aware of the collaboration than junior students (OR = 2.312; 95% CI = 1.541–3.468; *p* = 0.001). Female students were also more aware of the collaboration than males were (OR = 1.679; 95% CI = 1.170–2.410; *p* = 0.005) (Table 5). Among the students who reported awareness, 66% were aware of the link between dentistry and emergency medicine; 58%, of the link between dentistry and general surgery; and 57%, of the link between dentistry and otolaryngology. However, only 7% were aware of the association of dentistry with obstetrics & gynaecology (Fig. 1).

**Table 4:** Variables related to the students’ awareness toward medical-dental collaboration (chi-square test)

	Awareness, n (%)	*p*
Curriculum		0.001
Medicine (579)	458 (79)	
Dentistry (558)	521 (93)	
Year of study		0.001
Year 1–3 (721)	598 (83)	
Year 4–5 (416)	381 (92)	
Age, years		0.003
≤ 20 (754)	633 (84)	
> 21 (383)	346 (90)	
Gender		0.001
Male (332)	261 (78)	
Female (805)	718 (89)	
Having a family physician		0.837
Yes (907)	780 (86)	
No (230)	199 (87)	
Last dental check-up		0.007
< 1 year (21)	17 (81)	
≥ 1 year (406)	367 (90)	
No regular dental check-up (710)	595 (85)

**Table 5:** Variables related to the students’ awareness toward medical-dental collaboration (logistic regression)

Variable	Odds ratio	95% CI	*p*
Curriculum			0.001
Medicine ^Ref^			
Dentistry	3.352	2.243–5.010	
Year of Study			0.001
Year 1–3 ^Ref^			
Year 4–5	2.312	1.541–3.468	
Gender			0.005
Male ^Ref^			
Female	1.679	1.170–2.410	
Constant	2.190		0.001

*CI* confidence interval, *Ref* reference

## Discussion

Based on the findings in the present study, most students were of the opinion that oral health is an essential component of general health and that dentistry is an important field for medical practitioners. However, about one-third of the participants did not agree with medical-dental rotation (in both directions), but this is similar to the situation in another country [4]. This is probably because according to the curriculum of the Universitas Indonesia, medical students are not required to complete dental-related courses. Similarly, dental students have limited exposure to medical training. Nonetheless, both educators and researchers believe that medical-dental rotation is not only beneficial, but also essential for medical and dental education [14].

The results of this study revealed that the curriculum (medicine or dentistry) is related to the students’ attitudes toward and, also, awareness of medical-dental collaboration, as reported in previous studies [4, 15]. The dental program of the Universitas Indonesia emphasizes on problem-based learning (PBL), while the medical program is a mixture of discipline-based learning and PBL. As part of the PBL curriculum, students work in collaboration to solve the health problems of patients portrayed in case studies, to encourage intellectual exchange, to create a sense of personal involvement, and to inspire discoveries [14]. One study has reported that PBL can enhance the effectiveness of multi-professional collaboration [16]. Therefore, dental students with greater exposure to PBL may have more opportunities to develop good communication skills and a sense of collaboration, which will, consequently, influence their attitudes toward and awareness of medical-dental collaboration.

The year of study was also found to be related to the students’ attitudes toward and awareness of medical-dental collaboration, in accordance with the findings of prior studies [17, 18]. This can be explained in three ways. First, with advancement in a medical or dental program, the students’ sense of responsibility and teamwork are expected to improve [17]. Second, enhanced training and growth in knowledge and experience may lead to a better understanding of collaboration [18]. Third, students are exposed to various interprofessional collaborations during clinical training and as they advance through the program. Thus, greater exposure to clinical care and teamwork among senior students might explain why they have better awareness of medical-dental collaboration.

Another interesting finding of this study was that more female students, than males, reported that they were aware of collaboration. Recent evidence strongly suggests that team collaboration is greatly improved by the presence of females in the group [19]. Upon further examination, these effects can be explained in part by the higher levels of social sensitivity exhibited by females. Groups with more women also exhibited greater equality in conversation, thereby further enabling the group members to be responsive to one another and to make the best use of the knowledge and skills of other group members [19, 20]. Thus, on account of their higher social capabilities, female students may have greater interest in interprofessional collaboration.

In the present study, health behaviours such as having dental visits and having a family physician were correlated with awareness of and a favourable attitude towards learning and collaboration between medical and dental practice; this finding has been corroborated by a previous study [21]. Healthcare personnel’s positive attitude and adherence to good health behaviours not only affect their own health behaviour but also, potentially, influences the health behaviour of the patients and the community at large [22].

According to the current survey results, the most frequently reported medical disciplines correlated to dentistry were emergency medicine, general surgery, and otolaryngology. Here are three possible explanations for this. First, patients presenting with oral-maxillofacial trauma are commonly encountered in the Emergency Department. Medical students may consider dental students to be more equipped to deal with oral trauma as they have greater knowledge of oral-maxillofacial anatomy. Second, the nature of dental surgery is closely related to medical disciplines. Dentists, especially oral-maxillofacial specialists, perform surgeries for periodontal treatment, complicated extractions, and treatment of oral cancer. Third, the ears, nose, and throat are in the proximity of the oral cavity. These findings reveal that the students’ understanding of medical-dental collaboration is very shallow, when in fact, common oral diseases, such as dental caries and periodontal diseases, are reportedly related to various systemic diseases, such as respiratory diseases, cardiovascular diseases, diabetes, Alzheimer’s disease, and mental disorders (depression) [23, 24]. Once the depths of the links between medicine and dentistry can be elucidated further, students may understand better that medical-dental collaboration can be included in any discipline, as the need for such collaboration is very high.

From the findings of this study, we concluded that although students generally had a positive attitude toward and awareness of medical-dental collaboration in Indonesia, their understanding regarding the depth of this collaboration is very limited. It is important, and even vital in some circumstances, to improve health care services by enhancing close collaboration between medical doctors and dentists. Continuing interprofessional education could be another useful strategy to break down the stereotypes about other professionals and improve teamwork in clinical practice [25]. Such continuing education courses should emphasize on the importance of interprofessional collaboration and ensure that the knowledge of health care professionals is up to date, as this can increase their confidence when handling a problem, their ability to identify patient needs, and their suitability for specific referral cases [26, 27]. Medical and dental bodies can also contribute to medical-dental collaborations by developing guidelines for collaboration protocols, such as timing, indications, referral systems, etc. Healthcare professionals should also try to provide information about the relationship between oral health and general health to the public to arouse public awareness, as this might lead to a better understanding and acceptance of medical-dental collaboration by the patient.

All medical and dental students at the Universitas Indonesia were invited to participate in this study, in order to avoid selection bias. However, since non-probability sampling was used to select the study population, the results cannot be generalized for all the students in Indonesia. A web-based questionnaire was adopted in this study because of several advantages, such as lower cost, less time requirements, easier data management, and lower likelihood of missing data [28]. However, a meta-analysis reported that the response rate to a web-based survey was relatively low [29]. In this study, to achieve a desirable response rate, the coordinators of each class were given the responsibility of distributing the questionnaire to their class members and monitoring compliance, and the questionnaires were distributed in two waves. With this strategy, the response rate to this survey was relatively high. However, the number of non-respondents may have undermined the power of the study, so the response rate might still be a limitation to this study. On the other hand, there may have been a response bias because the participants may only represent those who have a positive disposition to the study objective. The questionnaire used simple distinctive dichotomous responses to measure attitude. Such an approach might be non-sensitive, decrease internal-reliability and force respondents to give simplistic responses to complex questions and, therefore, the results must be interpreted with caution [30]. Despite these limitations, the results of this survey offer valuable information about the current perspectives of Indonesian medical and dental students with regard to medical-dental collaboration, since no prior study has investigated this topic in Indonesia.

## Conclusion

In this study, medical and dental students were found to have a generally positive attitude toward and awareness of medical-dental collaboration. Specifically, dental students and senior students demonstrated a better attitude and more awareness than medical students and junior students, respectively. To further improve student attitudes and incorporate medical-dental collaboration into practice, the dental training program should emphasize on the addition of a medical training component into the curriculum. Additionally, further action should be taken to enhance the students’ understanding and knowledge of medical-dental collaboration, which is vital to improve resource efficiency and healthcare standards.

## Data Availability

The raw data are available from the authors to any author who wishes to collaborate with us.

## Abbreviation

PBL: Problem-Based Learning
